# Kidney derived apolipoprotein M and its role in acute kidney injury

**DOI:** 10.3389/fphar.2024.1328259

**Published:** 2024-01-19

**Authors:** Line S. Bisgaard, Pernille M. Christensen, Jeongah Oh, Federico Torta, Ernst-Martin Füchtbauer, Lars Bo Nielsen, Christina Christoffersen

**Affiliations:** ^1^ Department of Clinical Biochemistry, Copenhagen University Hospital—Rigshospitalet, Copenhagen, Denmark; ^2^ Department of Biomedical Sciences, University of Copenhagen, Copenhagen, Denmark; ^3^ Singapore Lipidomics Incubator, Life Sciences Institute, National University of Singapore, Singapore, Singapore; ^4^ Precision Medicine Translational Research Programme and Department of Biochemistry, Yong Loo Lin School of Medicine, National University of Singapore, Singapore, Singapore; ^5^ Department of Molecular Biology and Genetics, Aarhus University, Aarhus, Denmark; ^6^ The Faculty of Health Sciences, Aarhus University, Aarhus, Denmark

**Keywords:** apolipoprotein, apolipoprotein M, cell culture, HK-2 cell, transwell, kidney, acute kidney injury (AKI)

## Abstract

**Aim:** Apolipoprotein M (apoM) is mainly expressed in liver and in proximal tubular epithelial cells in the kidney. In plasma, apoM associates with HDL particles via a retained signal peptide and carries sphingosine-1-phosphate (S1P), a small bioactive lipid. ApoM is undetectable in urine from healthy individuals but lack of megalin receptors in proximal tubuli cells induces loss of apoM into the urine. Besides this, very little is known about kidney-derived apoM. The aim of this study was to address the role of apoM in kidney biology and in acute kidney injury.

**Methods:** A novel kidney-specific human apoM transgenic mouse model (RPTEC-hapoM^TG^) was generated and subjected to either cisplatin or ischemia/reperfusion injury. Further, a stable transfection of HK-2 cells overexpressing human apoM (HK-2-hapoM^TG^) was developed to study the pattern of apoM secretion in proximal tubuli cells.

**Results:** Human apoM was present in plasma from RPTEC-hapoM^TG^ mice (mean 0.18 μM), with a significant increase in plasma S1P levels. *In vitro* apoM was secreted to both the apical (urine) and basolateral (blood) compartment from proximal tubular epithelial cells. However, no differences in kidney injury score was seen between RPTEC-hapoM^TG^ and wild type (WT) mice upon kidney injury. Further, gene expression of inflammatory markers (i.e., IL6, MCP-1) was similar upon ischemia/reperfusion injury.

**Conclusion:** Our study suggests that kidney-derived apoM is secreted to plasma, supporting a role for apoM in sequestering molecules from excretion in urine. However, overexpression of human apoM in the kidney did not protect against acute kidney injury.

## Introduction

Human apolipoprotein M (hapoM) is a 21 kDa (non-glycosylated) or 25 kDa (glycosylated) apolipoprotein (Xu and Dahlback, 1999). Murine apoM (mapoM) is only detectable in the non-glycosylated form (Faber et al., 2004). ApoM in plasma is mainly associated with HDL particles and to a lesser extent with LDL and triglyceride-rich lipoproteins (Christoffersen et al., 2006). Approximately 5% of all HDL particles contain an apoM molecule (Christoffersen et al., 2006). ApoM is anchored to lipoproteins by a retained hydrophobic 21 amino acid signal peptide, which prevents rapid clearance of apoM in the kidneys (Christoffersen et al., 2008a). Thus, apoM does not circulate without association to lipoproteins in plasma.

ApoM is expressed in the liver and kidney in both humans (Zhang et al., 2003) and mice (Faber et al., 2004). The expression of apoM in the kidney is conferred primarily to the proximal tubular cells and to a lesser extent to the cells of the distal tubules and the glomeruli (Zhang et al., 2003). It is assumed that apoM in plasma is derived from the liver and due to its association with lipoproteins, apoM is not believed to be able to pass the healthy glomerular filtration barrier. Structurally, apoM contain a characteristic eight-stranded anti-parallel beta-barrel conformation surrounding a hydrophobic binding pocket (Duan et al., 2001). Sphingosine-1-phosphate (S1P), retinoic acids, and oxidized phospholipids have been identified as ligands for apoM (Ahnström et al., 2007; Christoffersen et al., 2011; Elsoe et al., 2012). At present, it is mainly the liver-derived plasma apoM/S1P-axis that has been investigated. Thus, studies have shown that the apoM/S1P-axis is necessary to maintain the endothelial barrier via S1P and the S1P receptors, play a role in lipid turnover and are involved in fibrosis formation (Christoffersen et al., 2011; Ding et al., 2016; Christoffersen et al., 2018; Ding et al., 2020; Hajny et al., 2021). Importantly, several studies have in addition indicated that besides being a transporter of small hydrophobic molecules, apoM might also modulate HDL particles and affect the turnover of apoB containing lipoproteins (Wolfrum et al., 2005; Christoffersen et al., 2008b; Kurano et al., 2013; Liu et al., 2014).

Due to the lack of appropriate animal models, the role of apoM specifically derived from the kidney is more speculative. *In vitro*, apoM binds the megalin receptor on proximal tubular cells and subsequently gets internalized (Faber et al., 2006), while apoM is undetectable in the urine from healthy wild type (WT) mice and humans (Faber et al., 2006). However, mice with kidney-specific megalin-deficiency loose apoM in the urine (Faber et al., 2006; Sutter et al., 2014). Therefore, it is hypothesized that kidney-derived apoM is secreted into the pre-urine where it binds S1P or other small molecules and subsequently gets endocytosed into the tubular cells via binding to megalin receptors. Thereby, apoM prevents urinary loss of S1P or other small hydrophobic molecules, which have been filtered in the glomeruli. This hypothesis is supported by a study with transgenic mice expressing human apoM without the signal peptide (Christoffersen et al., 2008a). These mice have undetectable levels of human apoM in plasma due to its rapid filtration in the kidney. However, human apoM was not found in the urine but was shown to be taken up by the proximal tubular cells in the kidney (Christoffersen et al., 2008a). Furthermore, apoM-deficient mice loose S1P in the urine (Sutter et al., 2014), but it is unknown whether this is due to lack of apoM in plasma or caused by lack of apoM in the kidney.

Besides megalin-deficiency, ischemia reperfusion (I/R) injury in rat kidneys also lead to rapid apoM excretion in urine (Xu et al., 2013). Further, knock out of S1P receptor 1 in proximal tubular epithelial cells leads to increased kidney injury upon both I/R and cisplatin induced acute kidney injury (Bajwa et al., 2010; Bajwa et al., 2015). This prompted the suggestion of kidney derived apoM as an important modulator of acute kidney injury.

To characterize the role of apoM on atherosclerosis and lipid metabolism, a panel of apoM gene-modified mice has been generated using the endogenous promoter (express apoM in liver and kidney) (Christoffersen et al., 2008b), the apoE promoter (express apoM primarily in liver) (Liu et al., 2014), and adenovirus (express apoM in liver) (Kurano et al., 2013). These models are not suitable for studying the specific function of kidney-derived apoM. Therefore, the purpose of this study was to generate a novel kidney-specific apoM transgenic mouse and subsequently to determine if kidney-derived apoM contributes to the plasma pool of apoM as well as its role in acute kidney injury.

## Materials and methods

### Animals

Mice were housed in a temperature-controlled facility with a 12-h light/dark cycle at the Panum Institute, University of Copenhagen, Denmark. They had free access to standard chow diet (Altromin 1314, Brogaarden) and water. Renal proximal tubule epithelial cell specific apoM-transgenic (RPTEC-hapoM^TG^) mice were backcrossed at least twice onto a C57BL6/J background for the basic characteristic of the model, while they were backcrossed at least three times for the injury studies. ApoM-transgenic Normal (apoM-Tg^N^) (Christoffersen et al., 2008b), apoM-transgenic High (apoM-Tg^H^) (Christoffersen et al., 2008b) and apoM knockout (apoM-KO) (Christoffersen et al., 2008b) mice were backcrossed >10 times. All animal experiments were approved by the Animal Experiments Inspectorate, Danish Veterinary and Food Administration, Ministry of Food, Agriculture and Fisheries, Denmark.

### Cloning and generation of kidney-specific human apoM transgenic mice

DNA containing the coding region of human apoM, 1729 bp 3′ to the polyA site and 3 bp 5′ to the ATG site was amplified from the Bacterial Artificial Chromosome (BAC) DNA (RP11-201G24, NCBI sequence AF129756, BACPAC) with PCR using Phusion HotStart High-Fidelity DNA Polymerase (F540, Thermo Scientific). Primer sequences were: 5′-CCG​CGG​ATA​CCG​TCG​ACC​CTG​AAG​ATG​TTC​CA-3′ and 5′-TCT​AGA​GAC​GGC​TTG​GTG​GCT​GGC​TA-3′. PCR products were purified with illustra GFX PCR DNA and Gel Band Purification kit (GE Healthcare), cloned into pCR-BluntII-TOPO (Life Technologies) and transfected into *E. coli* (*Escherichia coli)*. Plasmid DNA was isolated with QIAPrep Spin Miniprep kit (27106, Qiagen), digested with *SacII* and *SalI* and isolated on an agarose gel and purified with illustra GFX PCR DNA and Gel Band Purification kit (GE Healthcare).The Sglt2 promotor was isolated from pGEM-sglt2 5 pr-mut (Rubera et al., 2004) kindly provided by Dr. Isabelle Rubera by digestion with *SacII* and *SalI*, agarose gel electrophoresis and purification with illustra GFX PCR DNA and Gel Band Purification kit (GE Healthcare). The isolated fragment was ligated with the digested pCR-BluntII vector containing hapoM. The plasmid was transfected into *E. coli* and plasmid DNA was isolated with QIAPrep Spin Miniprep kit (27106, Qiagen). The correctness of the plasmid was assured by digestion with *ApaI* and sequencing of 700 bp surrounding the 5′ and 3′ end of human apoM and the 5′ end of Sglt2, respectively. The fragment containing the Sglt2 promotor and human apoM was isolated by digestion with *NsiI*, agarose gel electrophoresis and purification with illustra GFX PCR DNA and Gel Band Purification kit (GE Healthcare). Pronuclear injections of the isolated transgenes into fertilized mouse oocytes (B6D2F2 embryos) were done by Danish Genetically Modified Animal Resource, Aarhus University, Denmark, providing a novel mouse line with the following gene code: Tg(Sglt2-hsapoM)EMFU, and strain name: B6.D2-Tg(Sglt2-hsapoM)EMFU1.

### Genotyping

DNA was isolated and amplified from ear snips using REDExtract-N-Amp Tissue PCR Kit (XNAT, Sigma-Aldrich) according to the manufacturer’s protocol. Primers used were forward: 5′-GGG​ACT​TGA​ATT​CCT​CCA​CA-3′ and reverse: 5′-TGA​AGG​GAG​CAC​AGA​TCT​CA-3′. The primers span exon 3-5 of the human apoM gene. PCR products were analyzed with agarose gel electrophoresis.

### Kidney injury models

Mice were given a single injecting of cisplatin (20 mg/kg, *i.p.*) to induce kidney injury in RPTEC-hapoM^TG^ and WT mice. Following the injection, mice were monitored closely for the rest of the study, weighed once daily and given 1 mL of NaCl. 3 days after the cisplatin injection, a urine sample was collected, and mice were anesthetized with Zoletil (tiletamin 1.63 mg/mL, zolazepam 1.63 mg/mL, xylazin, 2.61 mg/mL, butorphanoltartrat 0.065 mg/mL) at a dose of 0.01 mL/g mouse. A blood sample was collected, and the mice were perfused with ice-cold saline after which the kidneys were excised and saved for histological and qPCR evaluation.

For I/R injury in RPTEC-hapoM^TG^, apoM-KO and WT mice, the right kidney was exteriorized via a dorsal incision. The blood vessels were exposed by carefully removing fat and connective tissue and the blood supply to the kidney was clamped at the renal pedicle for 30 min using a non-traumatic vascular clamp. During the procedure the kidney was covered with the skin and vet sterile gaze to avoid desiccation. After 30 min, the clamp was removed, and the kidney was gently pushed back into its retroperitoneal location. Mice were anesthetized with Zoletil during the procedure, and analgesia was administered by subcutaneous injection of buprenorphine (0.05 mg/kg mouse) during the procedure, and every 8 h after the procedure. 24 h of reperfusion was allowed after which a urine sample was collected, and mice were anesthetized. A blood sample was taken, mice were perfused with ice-cold saline and kidneys were saved for histological and qPCR evaluation.

### Blood samples and plasma measurements

Blood samples were taken either from the mandibular vein or retro-orbital (at termination of the mice). Blood was collected in EDTA microtubes and centrifuged at 3,500 rpm for 10 min at 4°C. Plasma urea and creatinine were measured with a Cobas1 8000 modular analyzer series (Roche A/S).

### Cell culture and transfection

HK-2 cells (ATCC, CRL-2190) were cultured on collagen coated wells (Type 1 Collagen, 5 μg/cm^2^ overnight, Sigma), in DMEM/F12 without phenol red (21041–025, Gibco) supplemented with 1 × GlutaMAX (Gibco), 1 × P/S (Gibco), 20 mM Hepes (Gibco), 25 ng/mL Hydrocortizone (H6909, Sigma) and 1 × ITS supplement (41400045, Gibco). 200 μg/mL Zeocin (45–0430, Invitrogen) was added for selection.

A stable cell line overexpressing human apoM (HK-2-hapoM^TG^) was established by transfecting the HK-2 cells with full length human apoM under the control of the human cytomegalovirus (CMV) promotor using the Flp-In system (Invitrogen). For experiments, cells were plated on collagen coated polycarbonate transwells (3401, Costar) and cultured until confluency. At day 4 after confluency was reached, cells were washed ones with PBS and the medium was changed to medium without FBS but with 0.05 mM BSA (A7030, Sigma).

### Transepithelial electrical resistance measurement

TEER was determined using an EndOhm Chamber 12G connected to an EVOM2 Meter (World Precision Instrument, England, United Kingdom). The measurements were done at room temperature. For data analysis, the resistance of an empty well was subtracted from the resistance in each of the wells and the data was adjusted for well size by multiplying with the well area of the insert.

### Permeability assay

Passive permeability (Papp) was determined using Cascade blue labelled 10 kDa dextrans (D1976, Invitrigen) and Fluorescein labelled 40 kDa dextrans (D1845, Invitrogen). The two dextrans were added directly to the apical wells for a final concentration of 0.5 mg/mL and 100 µL medium was collected from the basolateral (receiver) compartment at time point 15, 30, 45, 60, 90, 120, and 180 min. Each time the 100 µL medium was replaced with clean cell medium. At 180 min, a donor sample from the apical compartment was collected. During the procedure the plates were placed on a shaking table with a speed of 90 rpm, and in a controlled temperature of 37°C in atmospheric air between the sampling.

The receiver and the donor samples (diluted 1:100) as well as a control sample (0.5 mg/mL of each of the dextrans in clean medium) were pipetted to a black 96-well plate and the fluorescence signal was determined (EnSpire™ Multimode Plate Reader, PerkinElmer, Inc.; Cascade blue: excitation 400 nm, emission 440 nm; Fluorescein excitation 494 nm, emission 521 nm).

The apparent permeability (P_app_) was determined as previously described (Hubatsch et al., 2007; Tornabene et al., 2019). Briefly, Papp was calculated using the formula: P_app_ = J/(C_donor_*A), with J being the flux at steady state (µmol * sec^-1^), C_donor_ the initial concentration of the relevant marker molecule in the donor compartment (µM), and A the surface area of the filter (cm^2^). To determine J, the accumulation of the tested compound in the receiver chamber was plotted as a function of time and the slope in the linear region was determined.

### RNA extraction and gene expression analysis

RNA was extracted with TRIzol Reagent (15596–018, Life Technologies) according to the manufacturer’s protocol. cDNA was generated from 1 μg RNA using High-Capacity cDNA Reverse Transcription Kit (4368814, Applied Biosystems) and gene expression was determined by quantitative real-time PCR on TaqMan (Life Technologies). 18S, GAPDH, B2M and TBP was used as housekeeping (HK) genes.

Primer sequences:

Human apoM forward: 5′-GGG​ACT​TGA​ATT​CCT​CCA​CA-3′

Human apoM reverse: 5′-TGA​AGG​GAG​CAC​AGA​TCT​CA-3′

Mouse apoM forward: 5′-CCT​GGG​CCT​GTG​GTA​CTT​TA-3′

Mouse apoM reverse: 5′-CCA​TGT​TTC​CTT​TCC​CTT​CA-3′

Mouse TNFα forward: 5′-CTG​GCC​CAA​GGC​GCC​ACA​TC-3′

Mouse TNFα reverse: 5′-TGG​GGA​CCG​ATC​ACC​CCG​AAG-3′

Mouse ICAM forward: 5′-ATG​CCG​ACC​CAG​GAG​AGC​ACA​A-3′

Mouse ICAM reverse: 5′-TCG​ACG​CCG​CTC​AGA​AGA​ACC​A-3′

Mouse VCAM forward: 5′-CTT​CAT​CCC​CAC​CAT​TGA​AG-3′

Mouse VCAM reverse: 5′-TGA​GGA​GGT​CAG​GTT​CAC​AG-3′

Mouse IL-6 forward: 5′-ACA​AAG​CCA​GAG​TCC​TTC​AGA​GAG​A-3′

Mouse IL-6 reverse: 5′-GGC​ATA​ACG​CAC​TAG​GTT​TGC​CG-3′

Mouse S1P1 forward: 5′-TCG​CGC​GGT​GTA​CCC​AGA-3′

Mouse S1P1 reverse: 5′-CCA​GGC​AAA​CGC​TAG​AGG​GCG-3′

Mouse S1P2 forward: 5′-TTA​CTG​GCT​ATC​GTG​GCT​CTG-3′

Mouse S1P2 reverse: 5′-ATG​GTG​ACC​GTC​TTG​AGC​AG-3′

Mouse S1P3 forward: 5′-AAG​CCT​AGC​GGG​AGA​GAA​AC-3′

Mouse S1P3 reverse: 5′-TCA​GGG​AAC​AAT​TGG​GAG​AG-3′

Mouse 18s forward: 5′-CGC​GGT​TCT​ATT​TTG​TTG​GT-3′

Mouse 18s reverse: 5′-AGT​CGG​CAT​CGT​TTA​TGG​TC-3′

GAPDH forward: 5′-GTG​GTT​CAC​ACC​CAT​CAC​AA-3′

GAPDH reverse: 5′-GGT​GCT​GAG​TAT​GTC​GTG​GA-3′

B2M forward: 5′-TCT​CAC​TGA​CCG​GCC​TGT​AT -3′

B2M reverse: 5′-TTT​CAA​TGT​GAG​GCG​GGT​GG-3′

TBP forward: 5′-TAATCCCAAGCGATTTGCTGC-3′

TBP reverse: 5′-CTT​CAC​ATC​ACA​GCT​CCC​CA-3′

### Lipid analyses

Cholesterol in plasma samples and gel filtration chromatography fractions from both plasma and conditioned cell medium was analyzed enzymatically (11491458-216, Roche). Triglycerides were determined in plasma samples enzymatically according to the manufacturer’s protocols (TR0100, Sigma Aldrich).

### Gel filtration chromatography

Plasma samples were pooled (*n* = 2–4) and diluted 1:3.2 with PBS containing 0.01% EDTA and 500 μL were run at a Superose 6 10/300 GL column (17-5172-01, GE Healthcare). Fractions of 6 drops (∼280 μL) were collected. Protein profiles were determined by the absorbance read at 280 nm and cholesterol determined in individual fractions. Pools corresponding to the VLDL, LDL, and HDL peaks were collected for WB analysis of apoM.

Conditioned cell medium from respectively the apical (diluted 1:3) and the basolateral compartment was concentrated x6 using spin columns (10 k, Umicon Ultra, Merck) and pooled (medium from 3 wells). 500 μL of each sample were run at a Superose 6 10/300 GL column (17-5172-01, GE Healthcare) and fractions of 3 drops (∼140 μL) were collected. Also, a human plasma sample was run as reference material, and cholesterol determined in the fractions.

### Western blot

Gel filtration samples (15.6 μL) were run on 12% SDS-gels and blotted on PVDF membranes with iBlot Gel Transfer Device (Invitrogen). Membranes were blocked with 5% skim milk solution and incubated O/N with primary antibody at 4°C (rabbit anti-hapoM EPR2904, Genetex or goat anti-hapoM C54523, Life Span). After washing, membranes were incubated with secondary antibodies (anti-goat P0160, Dako or anti-rabbit P0448, Dako) for minimum 1 h at RT. Membranes were visualized with SuperSignal West Pico Chemiluminescent Substrate (34080, Thermo Scientific).

For urine samples, the analysis of mouse apoM (rabbit antibody EPR2904, Abcam) and mouse ApoA1 (K23001R, Biosite) were done as described above. The analysis of human apoM was done with an in-house apoM antibody (Christensen et al., 2017) directly labelled with IRDye 800CW High MW (LICOR). For this analysis membranes were blocked with Oddesey 927–40000 4°C O/N and visualized directly.

### Human apoM ELISA

Human apoM in plasma (10 μL) and urine (30 μL) samples from mice as well as conditioned medium (30 µL) and FPLC fractions (30 µL) of concentrated conditioned medium was analyzed with a specific human apoM ELISA as previously described (Bosteen et al., 2015). Briefly, samples were reduced with DTT and alkylated with iodoacetamide before addition to the sandwich ELISA. The monoclonal mouse antibody clone 1G9 (Abnova) was used as capture antibody and the rabbit antibody EPR2904 (Abcam) was used as detection antibody. Standard curve concentrations for the assay are 3.481, 2.660, 2.024, 1.369, 0.922, 0.695, 0.465, 0.234 nM. Urine samples were diluted 1:1000 while plasma samples were diluted 1:500 or 1:250. The apoM concentration detected is the same for dilution in water and urine.

### S1P extraction and quantification

Plasma S1P was determined as previously described (Burla et al., 2018). Briefly, lipids were extracted by mixing 20 µL of plasma with 200 µL butanol/methanol (1:1, v/v) solution containing an internal S1P d18:1 ^13^C2D2 standard (Toronto Research Chemicals, S681502), followed by derivatization of the lipids by a trimethylsilyl-diazomethane derivatization step (Narayanaswamy et al., 2014). Lipids were then separated on an Agilent 1290 UHPLC, using a Waters ACQUITY UPLC BEH HILIC, C18 (130 Å, 2.1 × 100 mm, 1.7 μm) column, and measured in an Agilent 6495 QQQ MS. The mobile phase A consisted of acetonitrile and 25 mM ammonium formate buffer (50:50, v/v) and mobile phase B was composed of acetonitrile and 25 mM ammonium formate buffer (95:5, v/v).

The Agilent MassHunter software was used to analyze the data, with lipid peaks being identified based on the specific precursor and product ion transitions, as well as the retention time. Finally, normalization to the internal standards was carried out as previously discussed (Burla et al., 2018).

### Histology

Kidneys were fixated in 4% formalin, embedded in paraffin, and 3 or 4 μm sections were collected and stained with PAS staining according to standard protocols.

Injury in both disease models was defined as: Tubular dilation, cell necrosis, loss of brush borders, protein casts formation. For the cisplatin model, kidney injury score was assessed by two blinded observers in at least 15 random pictures from the cortex area of the kidney taken from one section per mouse. Areas of kidney injury were marked on each picture using the Visiopharm software (Visiopharm, Denmark), and the values were then converted into a score from 0–4 (0 = no abnormalities, 1 = < 10% of picture affected, 2 = 11–25% of picture affected, 3 = 26–50% of picture affected, 4 = 51–75% of picture affected, 5 = > 76% of picture affected). For the I/R injury model, injury was assessed in 4 random selected pictures from the cortex area of both the injured and the control kidney taken from one section per mouse in a ×10 magnification. For each kidney a score from 1 to 3 was given, with 1 being small changes and 3 being major changes.

### Statistics

All statistical analyses, except analysis of qPCR data on kidneys from the I/R injury study, were performed using GraphPad Prism version 4.03 (GraphPad Software, Inc.). Groups of two were compared using Student’s t-test with welch correction or paired analysis when appropriate. Mann-Whitney test was used when one or two of the groups were not normally distributed. Groups of 3 were compared using one-way ANOVA with the Holm-Šídák *post hoc* test. *p*-values ≤0.05 were considered statistically significant. For analysis of qPCR data on kidneys from the I/R injury study, SPSS was used. Data was analyzed using mixed ANOVA.

## Results

### Generation of kidney-specific human apoM transgenic mice

A new transgenic mouse line only expressing human apoM in proximal tubular epithelial cells of the kidney, RPTEC-hapoM^TG^ mice, was generated by expressing human apoM under the control of the Sodium/glucose cotransporter 2 (Sglt2) promoter (Rubera et al., 2004). Six positive founders were generated and one founder with ∼38 genomic copies, as determined by real time PCR, was selected. The distribution between gender in the N3 generation contained equal numbers of positive and negative RPTEC-hapoM^TG^ males and females.

### Plasma apoM levels are increased in mice with kidney specific overexpression of apoM

**FIGURE 1:**
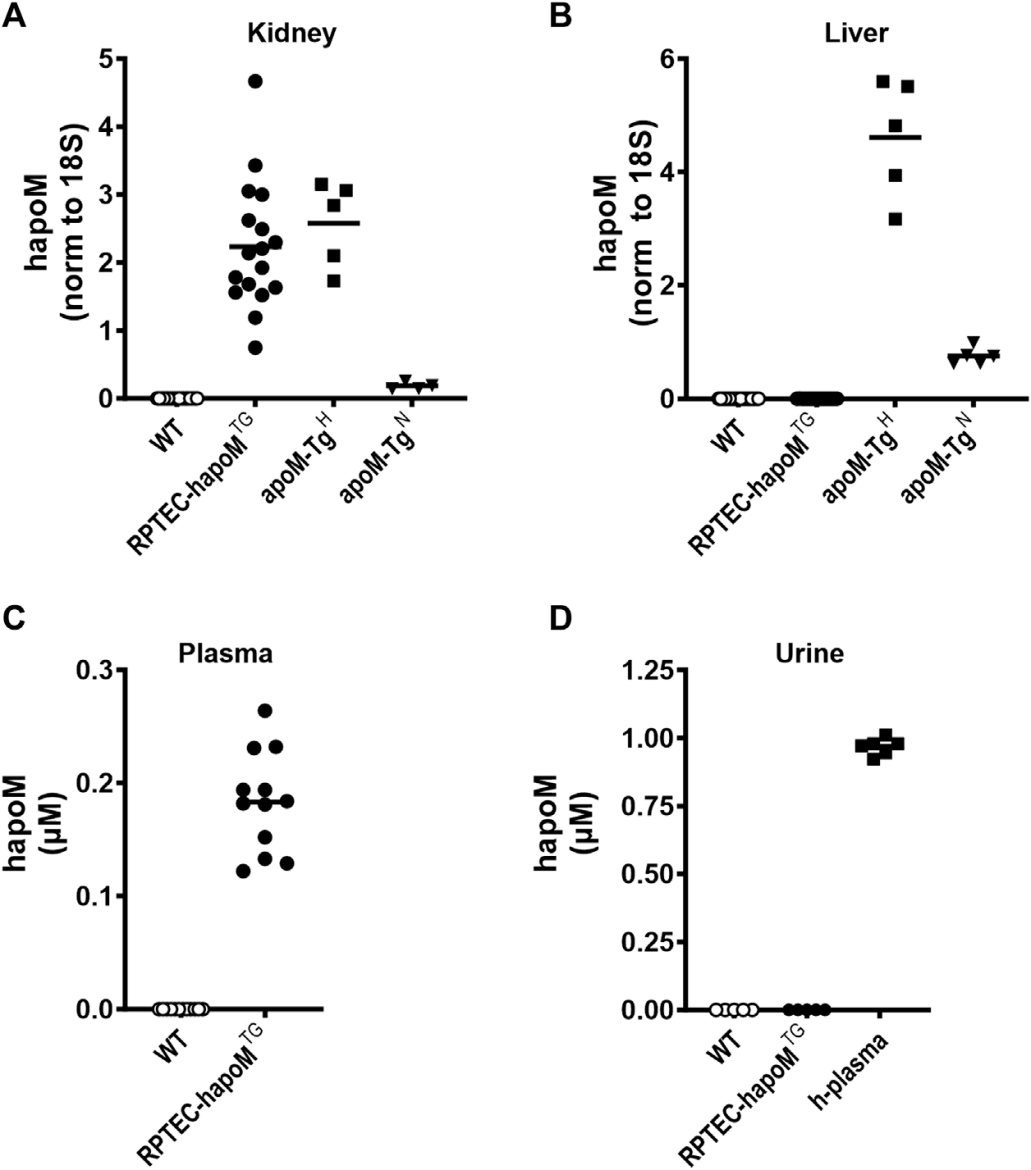
Expression of human apoM in liver and kidney, as well as plasma and urine concentration. Gene expression of human apoM were analyzed in kidney **(A)** and liver **(B)** from WT, PTEC-hapoM^TG^, apoM-Tg^H^ (10 times higher plasma apoM levels) and apoM-Tg^N^ (2 times higher plasma apoM levels) mice with qPCR and normalized to the expression of 18S. Human apoM in plasma from PTEC-hapoM^TG^ and their WT littermates **(C)** was measured with ELISA. Human apoM in urine samples from PTEC-hapoM^TG^, WT and human plasma samples **(D)** was measured with ELISA. For samples with an absorbance below the lowest standard in the standard curve, the concentration is noted as 0. Each symbol represents an individual mouse or human sample, and horizontal lines represent the mean.

Gene expression analysis of kidney and liver tissue revealed tissue-specific expression of human apoM in kidneys from RPTEC-hapoM^TG^ mice (Figure 1A), while there, as expected, was no expression in the liver (Figure 1B). The expression level of human apoM in kidneys from RPTEC-hapoM^TG^ mice was comparable to the expression levels seen in kidneys from apoM-Tg^H^ mice (mice with global overexpression of human apoM) (Figure 1A). Human apoM was undetectable in brain, heart, lung, skeletal muscle, duodenum, small intestine, colon, ventricle, ovaries, subcutaneous white adipose tissue, epididymal white adipose tissue, and interscapular brown adipose tissue in both lines (data not shown). Further, gene expression of endogenous mouse apoM was not affected by the overexpression of human apoM (Supplementary Figure S1).

No major phenotypes were observed in RPTEC-hapoM^TG^ mice. Weight-curves obtained over 10 weeks were identical for transgenic mice and their WT littermates (Supplementary Figure S2), no major differences were found in terms of organ weight or development between transgenic mice and WT littermates (Supplementary Figure S3), and there was no difference in the plasma concentration of urea (a marker for kidney injury) between RPTEC-hapoM^TG^ and WT mice (Supplementary Figure S4).

To test if kidney-derived apoM is secreted to plasma or is conserved in the kidney, plasma levels of human apoM were measured in RPTEC-hapoM^TG^ mice and WT littermates. Human apoM was present in plasma from RPTEC-hapoM^TG^ mice (range 0.12–0.26 μM, mean 0.18 μM), whereas human apoM as expected, was undetectable in WT littermates (Figure 1C). Further, human apoM was undetectable in urine samples from RPTEC-hapoM^TG^ mice and WT littermates (Figure 1D).

### Plasma S1P levels are increased in RPTEC-hapoM^TG^ mice

**FIGURE 2:**
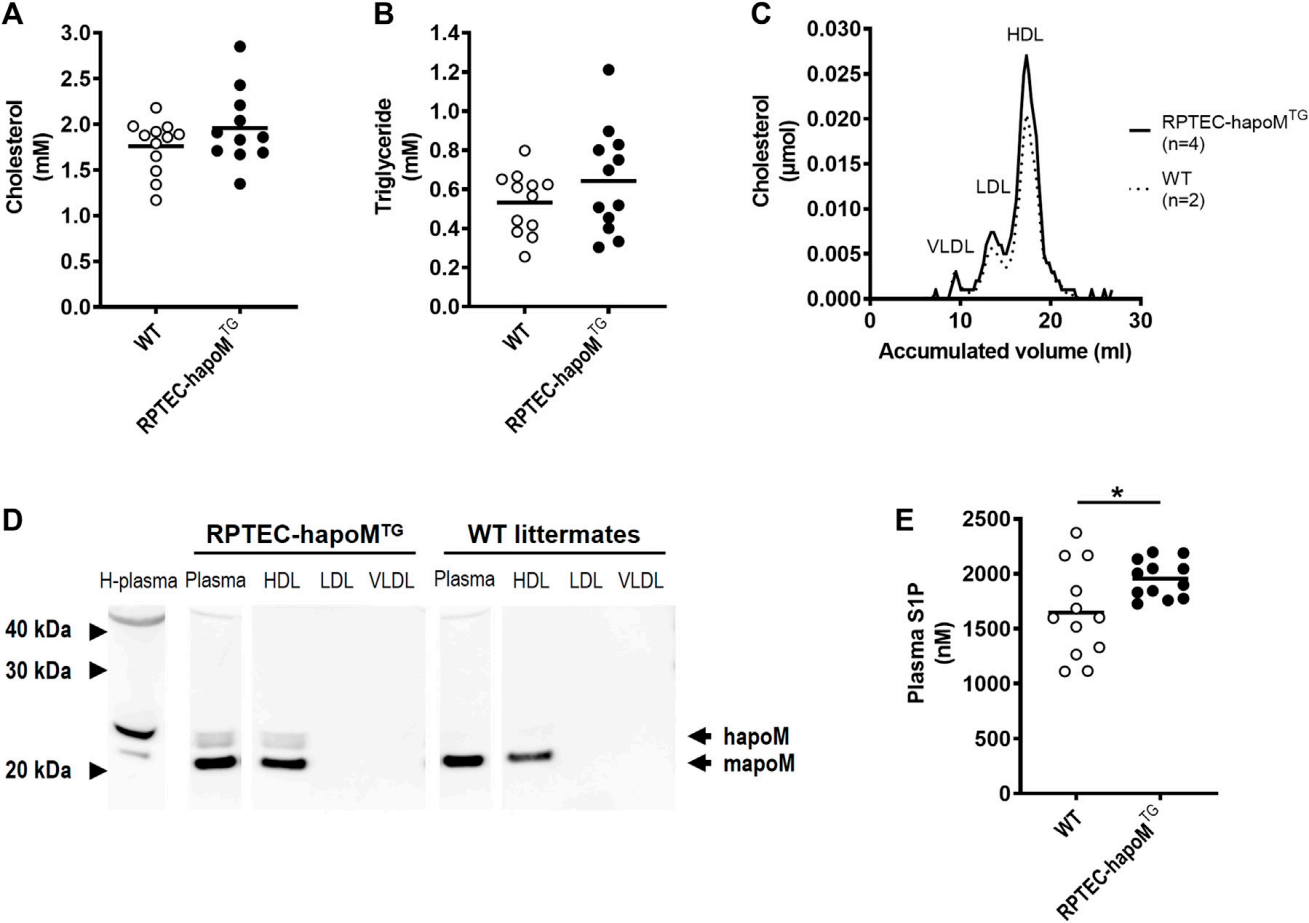
Characterization of the lipid profile and S1P levels in plasma. Plasma levels of cholesterol **(A)** and triglycerides **(B)** were measured in PTEC-hapoM (TG) and WT mice (littermates). Plasma from PTEC-hapoM^TG^ was analyzed by gel filtration chromatography and fractions were analyzed for cholesterol **(C)**. Pools of fractions corresponding to VLDL, LDL, and HDL (marked in C) were analyzed with Western blot against apoM **(D)**. Plasma S1P was analyzed by LC-MS **(E)**. Each point in A, B and E represents an individual mouse and lines represent the mean. Statistical analysis for A, B and E was done with Student’s t-test. *p < 0.05.

Global overexpression of apoM affects plasma lipids and lipoprotein metabolism (Christoffersen et al., 2008b). To address whether similar findings are present in the kidney specific overexpression model, the lipid profile of the mice was determined. Plasma levels of cholesterol and triglycerides were assessed in RPTEC-hapoM^TG^ mice and their WT littermates and the lipoprotein profile in plasma was determined by gel filtration chromatography. No difference was found in either total plasma cholesterol (Figure 2A), triglyceride concentration (Figure 2B) or lipoprotein composition measured as the cholesterol (Figure 2C) and protein (Supplementary Figure S5) profile. Normally, apoM is found exclusively in lipoproteins, where the majority is bound to HDL particles and smaller amounts are found in non-HDL particles. To test the distribution of human apoM among lipoproteins in RPTEC-hapoM^TG^ mice, pools corresponding to the VLDL, LDL, and HDL peaks from the gel filtration chromatography were analyzed with Western blot against human apoM (Figure 2D). Human apoM (∼25 kDa) was detectable in the HDL peak, while no human apoM was detectable in the LDL and VLDL fraction. In mice with a global overexpression of human apoM plasma, S1P is increased (Christoffersen et al., 2008b; Christoffersen et al., 2011). Interestingly, we found a significant increase in plasma S1P levels in the RPTEC-hapoM^TG^ mice compared to WT littermates (Figure 2E).

### ApoM is secreted both to the apical and basolateral compartment of proximal tubular epithelial cells

**FIGURE 3:**
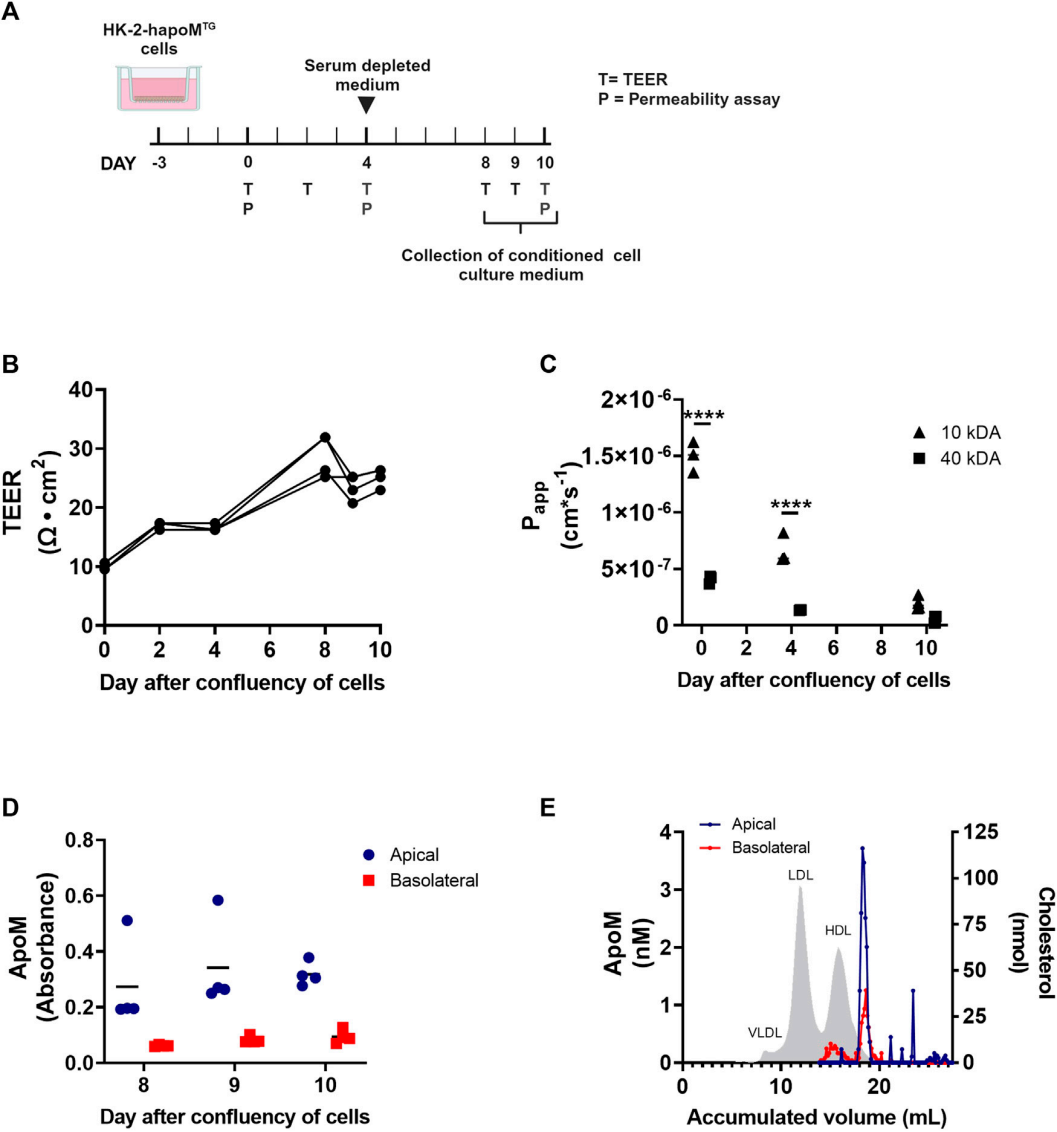
*In vitro* analysis of apoM secretion from proximal tubular epithelial cells. HK-2 cells overexpressing human apoM (HK-2-hapoM^TG^) was used to address the secretion of apoM to respectively the apical and basolateral compartment **(A)**. Teer measurement **(B)** as well as determination of permeability **(C)** was performed to confirm that the cells were confluent at day 8–10 after confluency. The apoM concentration in respectively the apical and basolateral compartment **(D)** was measured at day 8–10 after confluency. Medium from the apical and basolateral compartment was pooled, separated by FPLC and the apoM concentration in each of the fractions was determined **(E)**. Grey area depicts the cholesterol profile in human plasma. Each point in B-D represents an individual well (*n* = 3) and lines represent the mean. Statistical analysis for C was done using 2-way ANOVA with the Holm-Šídák *post hoc* test testing the difference between the two dextran sizes at each time point. Both the within factor (time) and the between factor (dextran size) was significant. ****p < 0.0001.

To address whether the human apoM measured in plasma is directly secreted to the blood compartment, or occur secondary to the secretion into the pre-urine, a transwell model of human proximal epithelial cells were established. A stable transfection of HK-2 cells with human apoM (HK-2-hapoM^TG^) was made and the cells were cultured on transwell inserts to collect conditioned medium from both the basolateral (comparable to blood) and apical compartment (comparable to pre-urine) (Figure 3A). To test the confluency of the cells, the resistance of the cell monolayer was tested at day 2, 4 8, 9, and 10 after confluency of the cells. As expected, the transepithelial electrical resistance (TEER) increased slowly from day 0–8 where it stabilized around 25 Ω × cm^2^ (Figure 3B). The passive permeability of fluorescence labelled dextrans was assessed at day 0, 4 and 10 after confluency. The permeability of both 10 kDA and 40 kDa dextrans decreased over time, with especially the permeability for 10 kDa dextrans being markedly reduced from day 0–10, suggesting establishment of a tighter monolayer (Figure 3C).

To evaluate the secretion pattern of apoM from proximal tubular epithelial cells, apoM concentration in respectively the apical and basolateral compartment, was determined at day 8, 9, and 10. ApoM was present in the conditioned medium from both the apical as well as the basolateral well, with the concentration being higher in the apical compartment (Figure 3D). ApoM is secreted with its hydrophobic signal peptide and thus needs to be associated with lipids in plasma. To elucidate what particles apoM is secreted with, the lipoprotein profile was determined using FPLC. Interestingly, we found that while apoM is only present in a very specific subfraction corresponding to small HDL particles in the apical compartment, apoM in the basolateral compartment were found on two distinct populations, one with a size similar to the one found in the apical compartment and one with a size similar to large HDL particles (Figure 3E).

### Overexpression of apoM in RPTECs does not protect against acute kidney injury

**FIGURE 4:**
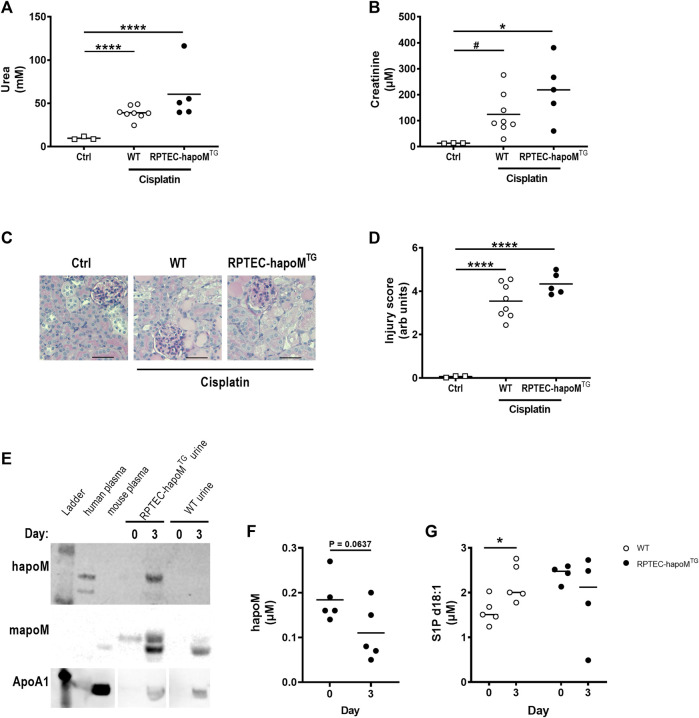
Characterization of kidney injury after cisplatin induced acute kidney injury. Plasma levels of urea **(A)** and creatinine **(B)** were measured in PTEC-hapoM^TG^, WT and ctrl mice. Kidney sections from PTEC-hapoM^TG^, WT and ctrl mice were stained with PAS stain **(C)**, representative pictures), and kidney injury was scores semi-quantitively **(D)**. Human apoM, mouse apoM and ApoA1 in urine from PTEC-hapoM^TG^ and WT mice was determined by Western blot before and after cisplatin injury **(E)**. The human apoM **(F)** and the S1P concentration **(G)** in plasma from PTEC-hapoM^TG^ and WT mice was analyzed before and after injury. Each point in A, B, D, F, and G represents an individual mouse and lines represent the mean. Statistical analysis for A, B and D was done using one-way ANOVA with the Holm-Šídák *post hoc* test. For F and G, statistics was done using paired Student’s t-test. *p < 0.05, ****p < 0.0001 ^#^ *p* < 0.05 analyzed by Student’s t-test with Welch’s correction.

Low apoM levels have been associated with increased fibrosis formation, hence we investigated whether overexpression of human apoM in proximal tubular epithelial cells would protect against kidney damage. Acute kidney injury was induced in the mice by a single injection of cisplatin inducing damage to the proximal tubular cells. To assess kidney injury, plasma urea and creatinine levels were measured 3 days post-injection. As expected, cisplatin injection significantly increased both urea and creatinine but there was no difference between RPTEC-hapoM^TG^ mice and their WT littermates (Figures 4A,B). Further, we performed histological evaluation of kidney injury on PAS-stained kidney sections (Figure 4C). Here we again found a significant increase in kidney injury after cisplatin injections but no difference between RPTEC-hapoM^TG^ mice and WT littermates (Figure 4D). No apoM was detected in urine before cisplatin injury (time 0) but both human and mouse apoM was present in urine at termination (Figure 4E). Further, we found a trend towards a reduction of human apoM in plasma upon cisplatin injury (Figure 4F). In contrast, the S1P concentration in plasma was increased for WT mice upon injury while no difference was found in the transgenic mice (Figure 4G).

**FIGURE 5:**
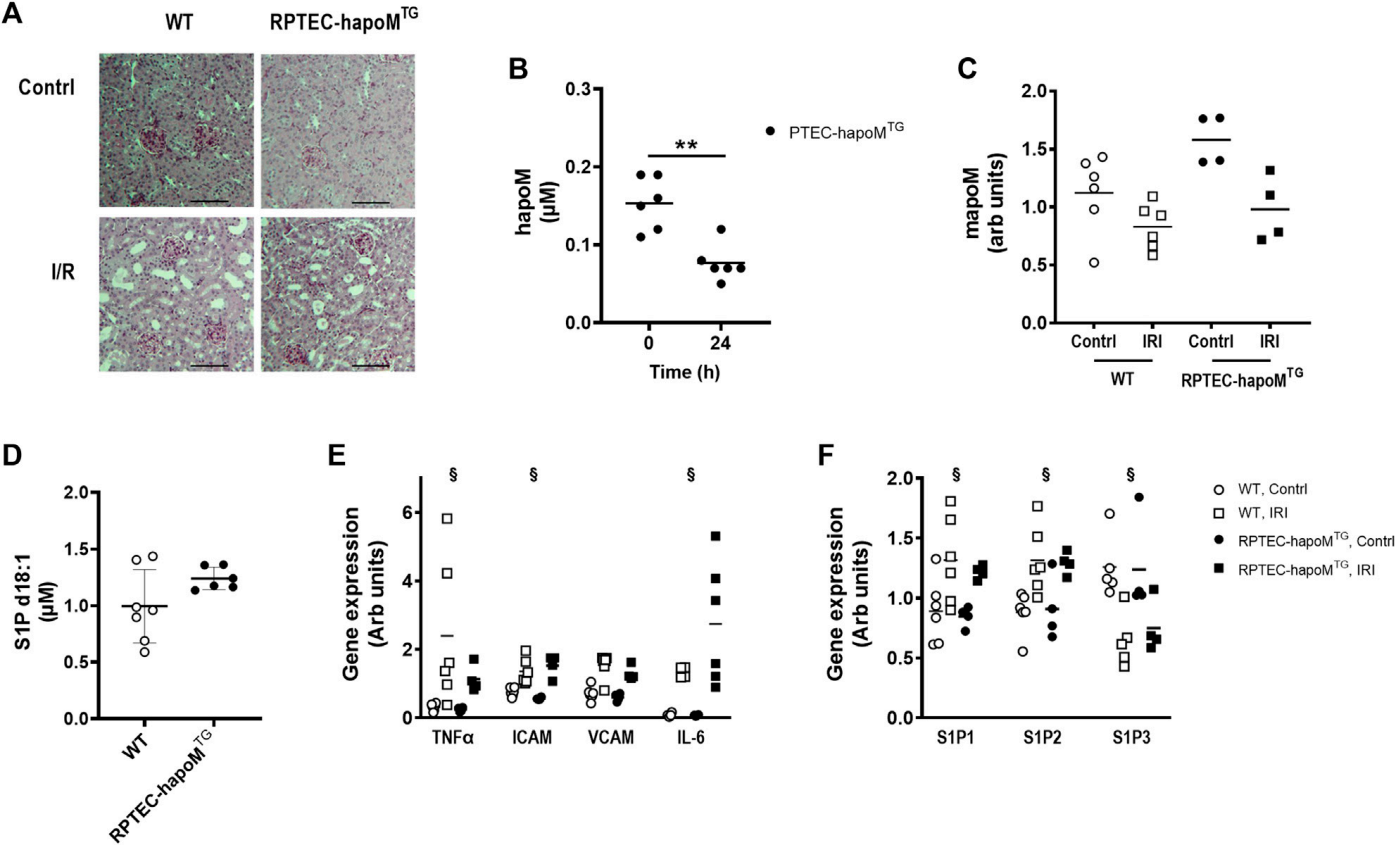
Characterization of kidney injury after I/R induced acute kidney injury. Kidney sections from both the injured and control kidney from PTEC-hapoM^TG^ and WT were stained with PAS. Representative pictures are shown in **(A)**. Plasma concentration of human apoM before and after IR injury was analyzed in PTEC-hapoM^TG^ by elisa **(B)**. Gene expression of mouse apoM was determined in both the kidney subjected to injury (IRI) and the contralateral control kidney (contrl) from PTEC-hapoM^TG^ and WT mice and normalized to M2B and TBP **(C)**. Plasma level of S1P d18:1 was determined after 24 h of reperfusion in both WT and PTEC-hapoM^TG^ mice **(D)**. Gene expression of inflammatory markers **(E)** as well as S1PR1-3 **(F)** was determined in both the kidney subjected to injury (IRI) and the contralateral control kidney (contrl) from PTEC-hapoM^TG^ and WT mice and normalized to verified housekeeping genes. Each point in B, C, D, and E represents an individual mouse and lines represent the mean. Statistical analysis for B was done with paired *t*-test, while mixed ANOVA was used for C, D and **(E)**. There were no interaction for any of the genes in either C, E or **(F)**. The within factor (injury) was significant for TNFα, ICAM, IL-6 and S1P1-3, while the between factor (genotype) was non-significant for all genes. **p < 0.05 ^§^ *p* < 0.05 for the withing factor (injury).

The effect of apoM on acute kidney injury was in addition assessed in a model of I/R injury. Kidney injury was induced by clamping of one kidney for 30 min followed by reperfusion for 24 h. The contralateral kidney served as control. As for cisplatin, no difference was found for either urea or creatinine between RPTEC-hapoM^TG^ mice and their WT littermates (data not shown). To address kidney injury, kidney sections were stained with PAS-staining (representative pictures are shown in Figure 5A). Histological evaluation of the kidneys revealed a significant injury of the tissue (e.g., tubular dilation and protein casts formation) in the kidney subjected to I/R injury compared to the contralateral control. However, we did not find any clear morphological differences between the injury seen in RPTEC-hapoM^TG^ mice and WT littermates (data not shown).

Plasma concentration of human apoM was significantly reduced after I/R injury (Figure 5B), as well as the gene expression of both human and mouse apoM in the kidney, without any difference between WT and RPTEC-hapoM^TG^ mice (Figure 5C). The S1P plasma levels between the two genotypes was unchanged.

To assess whether overexpression of human apoM affected the inflammatory status of the kidney, gene expression analysis on kidney tissue was performed. TNFα, ICAM and IL-6 were significantly increased in the kidney subjected to I/R injury compared to the contralateral kidney, with VCAM having the same trend (Figure 5E). However, no difference between RPTEC-hapoM^TG^ mice and their WT littermates was seen for any of the genes. We also assessed the expression level of genes involved in the apoM/S1P-axis (Figure 5F). Here we found that S1P receptor 1 and S1P receptor 2 were upregulated in the injured kidney compared to the control kidney while S1P receptor 3 was downregulated. However, overexpression of apoM did not affect gene expression of any of the analyzed genes involved in the apoM/S1P-axis.

To further address the role of apoM in acute kidney injury, gene expression of inflammatory markers as well as genes involved in the apoM/S1P-axis was determined in apoM-KO mice (mice with global deletion of apoM) subjected to I/R injury (Supplementary Figure S6). Similar to the results from RPTEC-hapoM^TG^ mice, ICAM and VCAM was significantly increased in kidneys subjected to I/R injury, with TNFα and IL-6 having the same trend. Further, S1P receptor 1 and 2 was upregulated while S1P receptor 3 was downregulated. For none of the genes was there an effect of genotype when comparing apoM-KO and WT mice.

## Discussion

ApoM is mainly produced by the liver and the proximal tubular epithelial cells in the kidney. While the role of liver derived apoM has been studied extensively, the role of kidney derived apoM is much more elusive. In this study, we have generated a novel transgenic mouse model with kidney-specific overexpression of human apoM. We show for the first time that kidney-derived apoM contributes to the plasma pool of apoM. This finding is supported by *in vitro* data showing that apoM is secreted to both the apical and basolateral compartment from proximal tubular epithelial cells. Finally, we show that overexpression of apoM locally in the kidney is not able to protect against either cisplatin or I/R induced acute kidney injury.

Unexpectedly, human apoM is detectable in plasma from RPTEC-hapoM^TG^ transgenic mice. This is highly important as it suggests that apoM is not only secreted to the apical (urine) site from the proximal tubular cells as earlier hypothesized, but also to the basolateral (plasma) compartment. It should be noted that, despite the overexpression of human apoM in this model, the concentration found is low compared to the apoM levels found in human plasma (∼0.9 µM) (Axler et al., 2007), suggesting that kidney-derived apoM is not a major contributor of plasma apoM levels. However, the presence of kidney-derived apoM in plasma raises a fundamental question of how and why kidney-derived apoM is transported from the proximal tubular cells into plasma.

In healthy individuals, apoM is not detectable in urine. Faber et al. have shown that apoM, in the pre-urine, is taken up by megalin-receptors in the proximal tubular cells, and secondly that megalin deficiency leads to apoM excretion in the urine (Faber et al., 2006). The apoM excreted into the urine is likely kidney-derived, since plasma-apoM is bound to lipoproteins, which are too large to undergo glomerular filtration. Once in the pre-urine, it has been suggested that apoM binds ligands, which have been filtered in the glomerulus. Subsequently, apoM transports them back to the proximal tubular cells, where they bind the megalin-receptor and are endocytosed into the proximal tubular cells (Faber et al., 2006). This hypothesis is supported by the finding that apoM without its signal peptide (and thus, without binding to HDL) are taken up by the proximal tubular cells (Christoffersen et al., 2008a). After endocytosis, at least two possible routes can explain how kidney-derived apoM is transferred to plasma. Either apoM bound to megalin can pass through the epithelial cells of the proximal tubule via transcytosis and enter the bloodstream on the basolateral side of the cell, or apoM are endocytosed after binding to megalin and subsequently degraded in the lysosomes. Newly formed apoM could then be transported to the basolateral side of the proximal tubular cells through the Golgi apparatus and be released to the bloodstream by exocytosis. Both processes have been described for retinol-binding protein (Christensen et al., 1999; Marino et al., 2001), which is structurally and functionally related to apoM. In this paper, we find that apoM secreted to the apical side of the proximal tubular cells associates to particles with a relative defined size that is smaller than HDL, similar to what is found by Faber et al., 2006. In contrast, the particles at the basolateral side associated with apoM seem to have two distinct sizes, one similar to the size found in the apical compartment and another with a broader size span in the HDL size range. This finding at least suggest that the secretion pathway for apoM differs between the apical and basolateral compartment, or that the particles are somehow modified before secretion to the basolateral side. Further studies are however needed to reveal the precise secretion pathway.

In plasma, apoM serves as an important carrier of S1P and plays a significant role in vascular barrier functions, lipid turnover, and fibrosis (Christoffersen et al., 2011; Ding et al., 2016; Christoffersen et al., 2018; Ding et al., 2020; Hajny et al., 2021). The role of apoM in the kidney is less explored. One possible role of kidney-derived apoM is to help preserve glomeruli-filtered ligands. In the pre-urine apoM could bind S1P or other small molecules such as retinoic acids or oxidized phospholipids and assist re-uptake of ligands into the proximal tubular cells. The hypothesis is supported by findings from Sutter et al. who measure S1P in the urine from apoM-deficient mice (Sutter et al., 2014), and now also by our data showing that plasma S1P levels are increased in mice with kidney specific overexpression of human apoM. However, proximal tubular epithelial cells are also able to *de novo* synthesize S1P (Blanchard et al., 2018). The increased S1P levels found in our study could therefore also be a result of an increased secretion of endogenously produced S1P with apoM from the proximal tubular cells in the kidney. Further studies will be needed to unravel this. At this point it is also not clear whether apoM might sequester other molecules than S1P from the urine. This would again be important to address to fully elucidate the role of kidney derived apoM.

A number of studies have shown that modulation of S1P signaling affects the outcome after acute kidney injury. Thus, stimulation with FTY720 (an S1P analogue) or SEW-2827 (specific S1P receptor 1 agonist) leads to less injury after both cisplatin and I/R injury (Awad et al., 2006; Bajwa et al., 2010; Bajwa et al., 2015). Further, kidney injury induced by both cisplatin and I/R injury are increased in mice with local knockout of the S1P receptor 1 in proximal tubular epithelial cells (Bajwa et al., 2010; Bajwa et al., 2015). A recent publication suggest that the detrimental effects of IL-1 signaling during acute kidney injury are mediated via suppression of apoM expression in proximal tubular epithelial cells highlighting the possible important role of apoM in kidney functionality (Ren et al., 2023). To our knowledge the present study is, however, the first to look directly at the role of apoM locally produced in the kidney on acute kidney injury. Despite the increased plasma S1P levels we see in the naïve RPTEC-hapoM^TG^ mice, we do not find any protective effect of kidney specific overexpression of apoM on the degree of kidney injury. Why this is the case when S1P modulation does protect against kidney injury, is at this point elusive, but one explanation might be that the S1P increase is relatively modest or the impact of S1P via the family of S1P-receptors are counteracted. Similar, our observations in apoM-KO mice support that a change in apoM levels does not affect inflammatory markers after I/R induced acute kidney injury.

It is here, however, also important to notice that increased S1P levels are not always protective. Thus, mutations in the sphingosine phosphate lyase gene, the enzyme that mediates the degradation of S1P, have been shown to result in development of severe illness including risk of kidney failure, suggesting that accumulation of S1P intracellularly is critical (Janecke et al., 2017; Lovric et al., 2017; Prasad et al., 2017)*.* A possible explanation for the lack of effect of apoM overexpression on kidney injury observed in the present study, could therefore be that locally produced apoM levels in proximal tubuli cells sequesters S1P intracellularly followed by detrimental intracellular effects. Such a hypothesis will need further investigation.

Some limitations need to be mentioned. In both kidney injury models, we did see a decrease in apoM levels, both in plasma and in the kidney tissue. This might decrease any protective effects of the overexpression of apoM. Also, despite the overexpression nature of the mouse model, we only see modest increases in plasma apoM levels. A more potent increase in plasma apoM levels, could still be protective.

In conclusion, we have developed a unique mouse model with overexpression of apoM in the proximal tubular epithelial cells. With this model we have shown that apoM can be secreted to the basolateral site and thus into the blood compartment in the kidney, which was confirmed *in vitro*. Further, we find that the overexpression of apoM also results in increased S1P plasma levels. The increased apoM levels do, however, not protect against acute kidney injury.

We believe that this unique mouse model will be highly valuable for future studies exploring the role of apoM/S1P in the kidney, both in the normal setting and in pathophysiological conditions such as chronic kidney injury, where only a few studies so far have looked at the role of apoM and none of these have addressed the specific role of kidney-derived apoM.

## Data Availability

The raw data supporting the conclusion of this article will be made available by the authors, without undue reservation.

## Abbreviations

apoM, apolipoprotein M; hapoM, human apoM; mapoM, mouse apoM; apoM-Tg^H^, apoM-transgenic High; apoM-Tg^N^, apoM-transgenic Normal; apoM-KO, apoM knockout; HDL, high density lipoprotein; LDL, low density lipoprotein; S1P, sphingosine-1-phosphate; Sglt2, sodium/glucose cotransporter 2; RPTEC-hapoM^TG^, renal proximal tubule epithelial cell specific apoM-transgenic mice; WT, wild type; I/R, ischemia reperfusion; HK-2-hapoM^TG^, HK-2 cells with stable overexpression of hapoM; Papp, Passive permeability.
