# Supplementary material for: Kidney derived apolipoprotein M and its role in acute kidney injury
Source: Front Pharmacol. 2024 Jan 19;15:1328259. doi: 10.3389/fphar.2024.1328259 (PMC10834784; doi:10.3389/fphar.2024.1328259)
Supplement: Supplementary file 1 [file DataSheet1.docx]

**Supplementary Data**

This material provides additional information for the article:

**Kidney derived apolipoprotein M and its role in acute kidney injury**

Line S Bisgaard^1,2^, Pernille M Christensen^1,2^, Jeongah Oh^3^, Federico Tesio Torta^3,4^, Ernst-Martin Füchtbauer E^5^, Lars Bo Nielsen^6^, and Christina Christoffersen^1,2^

^1^Department of Clinical Biochemistry, Copenhagen University Hospital, Rigshospitalet, Denmark

^2^Department of Biomedical Sciences, University of Copenhagen, Denmark

^3^Singapore Lipidomics Incubator, Life Sciences Institute, National University of Singapore, Singapore 117456, Singapore.

^4^Department of Biochemistry, Yong Loo Lin School of Medicine, National University of Singapore, Singapore 117596, Singapore.

^5^Department of Molecular Biology and Genetics, Aarhus University, Denmark

^6^The Faculty of Health Sciences, Aarhus University, Denmark.

**Corresponding author:**

Christina Christoffersen, MD, PhD, DMSc

Address: Department of Clinical Biochemistry, Copenhagen University Hospital - Rigshospitalet, Blegdamsvej 9, 2100 Copenhagen, Denmark

Phone: +45 3545 3007 Fax: +45 3545 2880

E-mail: christina.christoffersen@regionh.dk

**
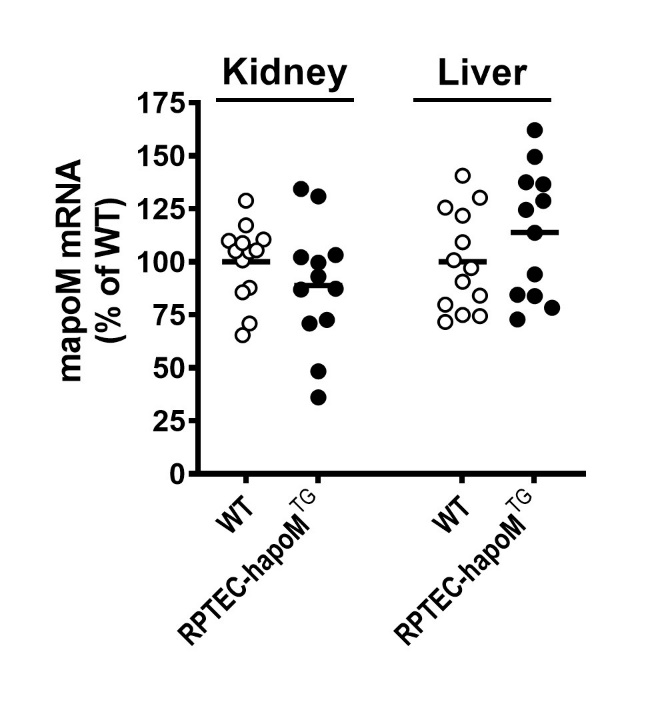
**

**Suppl. Fig. 1. Gene expression of mouse apoM in kidney and liver.**

Gene expression of mouse apoM in kidney and liver from WT and RPTEC-hapoM^TG^ mice was analyzed with quantitative RT-PCR. Gene expression is normalized to the expression of 18S. Each symbol represents an individual mouse and horizontal lines represent the mean. Data is presented as % of WT for respectively kidney and liver.

**
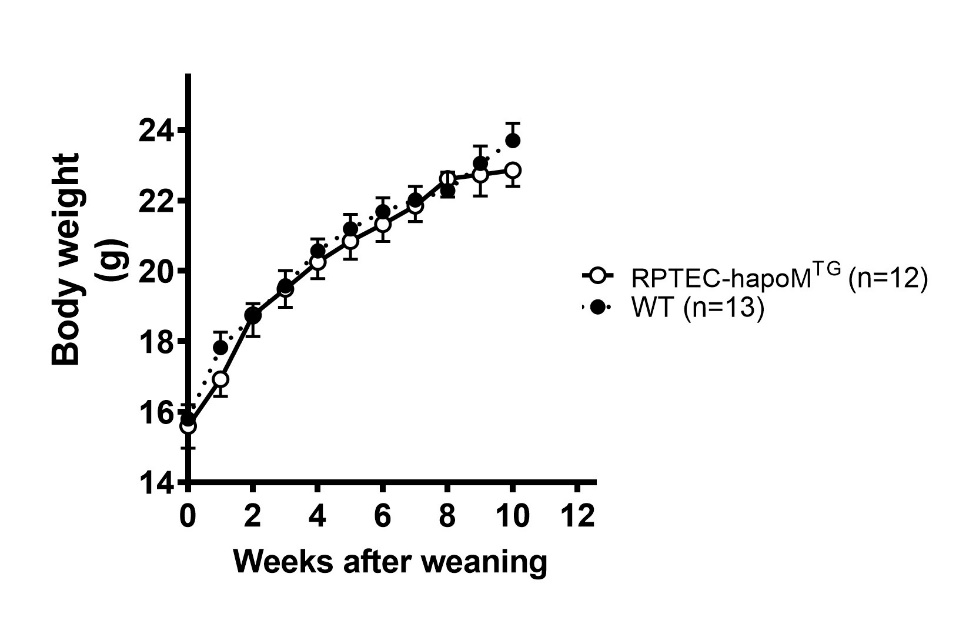
**
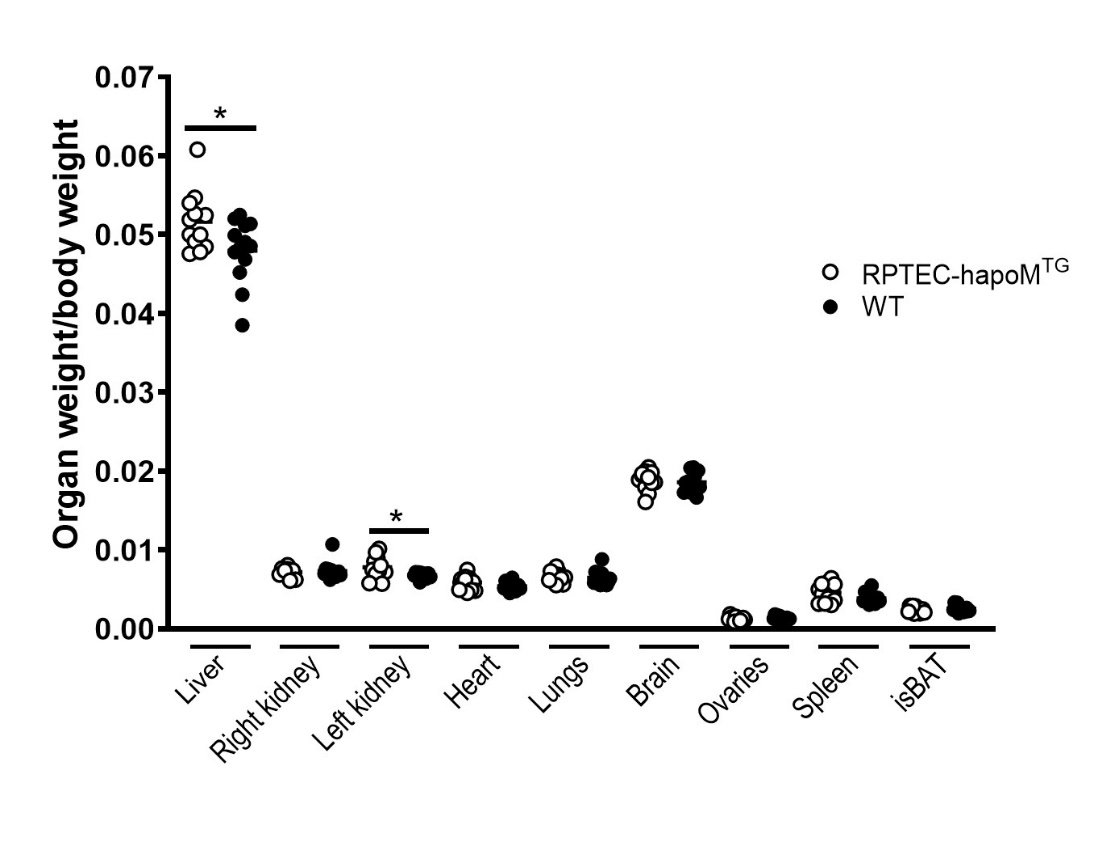


**Suppl. Fig. 2. Mouse weight.**

Mice were weighted at weaning (4-6 weeks of age) and the following 10 weeks. Each point represents the mean±SEM. Number of mice in each group is indicated in parentheses.

**Suppl. Fig. 3. Organ weight.**

Organ weights were determined in 20-weeks old RPTEC-hapoM^TG^ (n=12-13) and WT (littermates) and normalized to body weight. Each point represents an individual mouse and lines represent the mean. isBAT=interscapular brown adipose tissue. Statistics were done with Student's t-test for each organ. *p≤0.05.

**
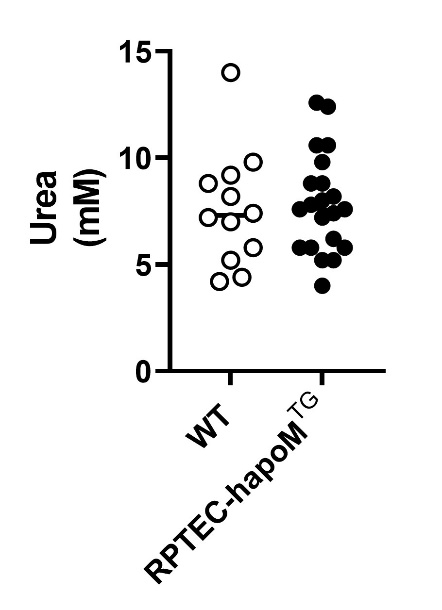
**


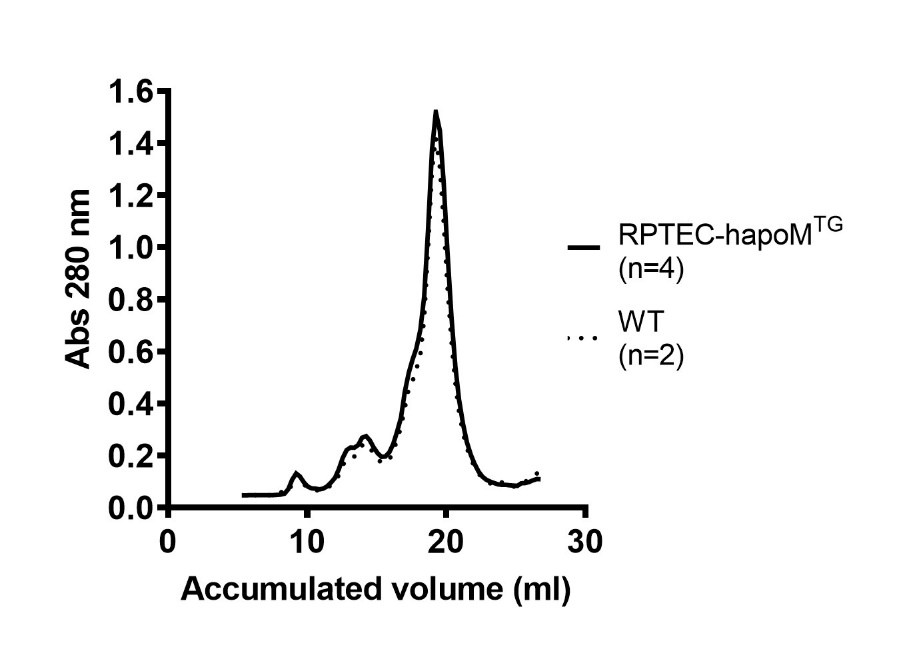


**Suppl. Fig. 4. Plasma concentration of urea in naïve WT and RPTEC-hapoM^TG^ mice.**

The plasma concentration of urea was determined in naïve WT and RPTEC-hapoM^TG^ mice (n=12-21). Each point represents an individual mouse and lines represent the mean. Statistics were done with Student's t-test. * p≤0.05.

**Suppl. Fig. 5. Plasma protein profile in RPTEC-hapoM^TG^ and WT mice.**

The protein profile was determined in fractions from gel filtration chromatography of plasma from RPTEC-hapoM^TG^ and WT mice.


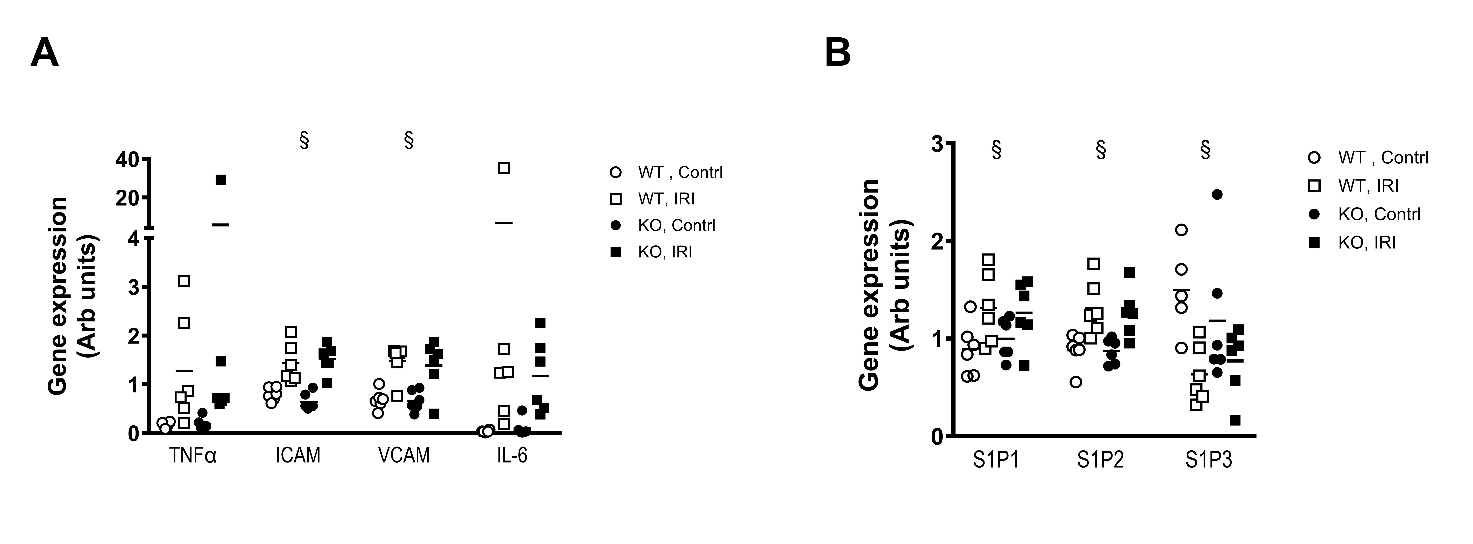


**Suppl. Fig. 6. Gene expression in kidneys from apoM-KO and WT mice after I/R induced acute kidney injury.**

Gene expression of inflammatory markers (A) as well as S1PR1-3 (B) was determined in both the kidney subjected to injury (IRI) and the contralateral control kidney (contrl) from apoM-KO and WT mice and normalized to verified housekeeping genes.

Each point in A and B represents an individual mouse and lines represent the mean. Mixed ANOVA was used for the statistical analysis. There were no interaction for any of the genes in either A or B. The within factor (injury) was significant for ICAM, VCAM and S1P1-3, while the between factor (genotype) was non-significant for all genes.

^§^p<0.05 for the withing factor (injury)
